# Characterisation and differential diagnosis of neurological complications in adults with phenylketonuria: literature review and expert opinion

**DOI:** 10.1007/s00415-023-11703-4

**Published:** 2023-04-20

**Authors:** Martin Merkel, Daniela Berg, Norbert Brüggemann, Joseph Classen, Tina Mainka, Simone Zittel, Ania C. Muntau

**Affiliations:** 1 Endokrinologikum Hamburg, Lornsenstraße 6, 22767 Hamburg, Germany; 2 Asklepios Campus Hamburg, Semmelweis University, Hamburg, Germany; 3 Department of Neurology, Christian-Albrechts University, Kiel, Germany; 4 Department of Neurology, University of Lübeck, Lübeck, Germany; 5 Department of Neurology, Leipzig University Medical Center, Leipzig, Germany; 6 Department of Neurology, Charité University Medicine Berlin, Berlin, Germany; 7 Berlin Institute of Health at Charité Universitätsmedizin Berlin, BIH Biomedical Innovation Academy, BIH Charité Clinician Scientist Program, Berlin, Germany; 8 Department of Neurology, University Medical Center Hamburg-Eppendorf, Hamburg, Germany; 9 University Children’s Hospital, University Medical Center Hamburg-Eppendorf, Hamburg, Germany

**Keywords:** Adult, Diagnosis, Neurological signs and symptoms, Phenylketonuria, PKU

## Abstract

**Objective:**

Phenylketonuria (PKU) is a rare inherited metabolic disorder characterised by elevated phenylalanine (Phe) concentrations that can exert neurotoxic effects if untreated or upon treatment discontinuation. This systematic review supported by expert opinion aims to raise awareness among the neurological community on neurological complications experienced by adults with PKU (AwPKU).

**Methods:**

The PubMed database was searched for articles on neurological signs and symptoms in AwPKU published before March 2022. In addition, two virtual advisory boards were held with a panel of seven neurologists and two metabolic physicians from Germany and Austria. Findings are supported by three illustrative patient cases.

**Results:**

Thirty-nine articles were included. Despite early diagnosis and treatment, neurological signs and symptoms (e.g. ataxia, brisk tendon reflexes, tremor, visual impairment) can emerge in adulthood, especially if treatment has been discontinued after childhood. In PKU, late-onset neurological deficits often co-occur with cognitive impairment and psychiatric symptoms, all of which can be completely or partially reversed through resumption of treatment.

**Conclusion:**

Ideally, neurologists should be part of the PKU multidisciplinary team, either to bring lost to follow-up patients back to clinic or to manage symptoms in referred patients, considering that symptoms are often reversible upon regaining metabolic control. The current findings have been combined in a leaflet that will be disseminated among neurologists in Germany and Austria to create awareness.

**Supplementary Information:**

The online version contains supplementary material available at 10.1007/s00415-023-11703-4.

## Introduction

Neurological deficits are commonly reported in late-diagnosed patients with phenylketonuria (PKU), a rare inborn error of metabolism caused by autosomal recessive mutations in the phenylalanine hydroxylase (*PAH*) gene [1, 2]. Deficiency of PAH prevents the conversion of phenylalanine (Phe) into tyrosine, inducing a state of hypotyrosinemia along with elevated blood Phe concentrations that compete with large neutral amino acids at the L-type amino acid carrier (LAT1) at the blood–brain barrier [2, 3]. Through disturbed monoamine neurotransmitter and cerebral protein synthesis, untreated patients with PKU are prone to develop significant developmental delay and severe neurological disability [3]. Since the implementation of newborn screening programmes for PKU, most patients are treated shortly and continuously after birth, preventing severe complications by restricting the intake of dietary Phe with or without supplementation of sapropterin dihydrochloride [2, 4]. Despite early diagnosis, neurological manifestations can still present in adulthood, mainly because a substantial proportion of early treated adult patients have blood Phe concentrations above guideline-recommended target ranges (European target: 120–600 µmol/L [2]; United States [US] target: 120–360 µmol/L [4]) [5, 6]. The complexity and burden of the Phe-restricted diet are the main reasons for the deteriorating treatment adherence, which usually starts in late childhood [7, 8]. Although current European and US guidelines recommend lifelong treatment [2, 4], relaxation of the Phe-restricted diet in adulthood is common practice in Germany due to the former recommendation to maintain blood Phe concentrations below 1200 µmol/L after the age of 15 years [9]. In some cases, treatment discontinuation coincides with declining in-clinic attendance [5]. Besides the burden of treatment, the historical focus of PKU care on the paediatric population and absence of paediatric-to-adult-care transition programmes have substantially contributed to lost to follow-up numbers that are reported to be as high as 50% of the adult PKU population, resulting in a significant number of patients with poor metabolic control in adulthood who are also prone to develop nutritional deficiencies [5, 10–13].

To manage the neurological symptoms in both late-diagnosed and early treated adults with PKU (AwPKU), neurologists should be involved in the multidisciplinary PKU treatment team when needed. However, neurologists may lack experience in rare inherited metabolic disorders and may not immediately recognise PKU as the underlying cause of neurological deficits. This manuscript provides an overview of PKU-associated neurological signs and symptoms with a focus on early treated adult patients. It aims to raise awareness among the neurological community to ultimately improve the management of patients with PKU who may present with neurological complications in adulthood and may or may not have discontinued follow-up.

## Materials and methods

### Systematic literature review

A systematic literature search of MEDLINE was conducted in March 2022 using the PubMed search engine to identify relevant studies. Inclusion and exclusion criteria were defined according to the PICOS strategy:

- Population: AwPKU (defined as ≥ 18 years of age).
- Interventions: Phe-restricted diet, pharmacological treatment or no treatment.
- Comparator: none, between groups or non-PKU controls.
- Outcomes: prevalence and severity of neurological symptoms.
- Study types: randomised controlled trials (RCTs), non-RCTs, observational studies, case series and systematic literature reviews (SLRs)/meta-analyses.

Keywords for the literature search were phenylketonuria, hyperphenylalaninemia, phenylalanine hydroxylase deficiency, adults, adulthood, neurologic, neurological, tremor, ataxia, seizure, parkinsonism, paraparesis and brisk tendon reflexes. Non-human studies, review articles, letters, conference abstracts, single case reports, position papers, author replies and non-English articles were excluded. SLRs and meta-analyses on the topic were reviewed to identify any additional relevant studies. Retrieved papers were screened to select those reporting clinical data on neurological symptoms among AwPKU. Duplicates and papers not meeting the inclusion criteria were removed from the literature review.

### Expert meetings

A 4-h virtual advisory board was held with seven neurologists and one metabolic clinician from Germany and Austria, discussing the spectrum of neurological signs and symptoms experienced by AwPKU. During the advisory board, a leaflet was developed that summarises the most common neurological signs and symptoms in AwPKU, providing recommendations for the differential diagnosis. Except for two neurologists, all experts participated in a follow-up advisory board held on a secure online platform to which an additional metabolic clinician with PKU experience contributed. During the follow-up advisory board, additional expert opinion was collected together with the development of five statements, which were revised in two voting rounds until consensus was reached among all experts.

### Patient cases

Information on three patient cases was provided by the Endokrinologikum Hamburg, Germany, part of the Amedes group. Participants gave written consent for online publication and dissemination of the anonymised patient information.

## Results

### Systematic literature review

**Fig. 1:**
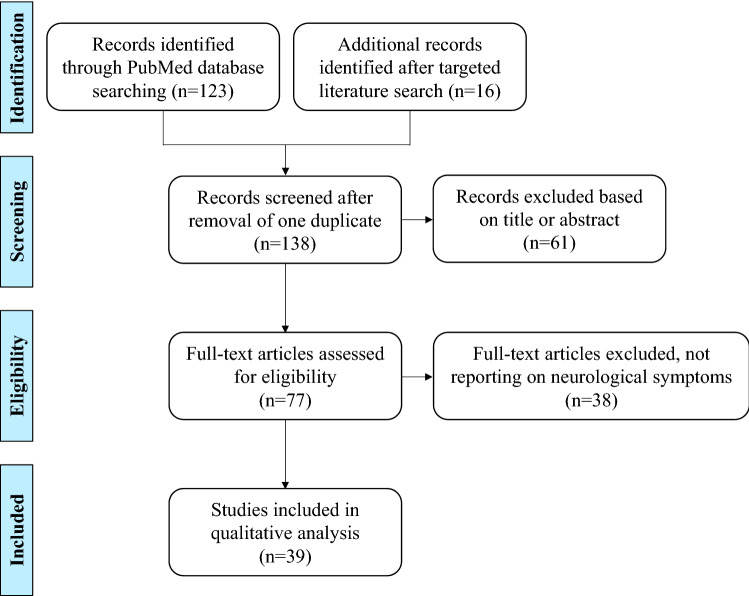
Study selection process according to Preferred Reporting Items for Systematic Reviews and Meta-Analysis (PRISMA) flowchart

Of the 139 retrieved articles, 39 met the inclusion criteria, including 17 observational studies (3 case–control studies, 6 cohort studies, 8 cross-sectional studies), 20 case series and 2 SLRs (Fig. 1). Full tables of the results, excluding the two retrieved SLRs, are presented in the Supplementary Information. Findings of the SLR are summarised in the sections below and supported by expert opinion if applicable.

### Spectrum of neurological signs and symptoms

#### Neurological signs and symptoms in early treated AwPKU

**Table:** 

**Despite early diagnosis and treatment, neurological signs and symptoms may develop in adults with PKU upon treatment discontinuation or in patients who are not able to achieve sustained metabolic control**	

It is well established that AwPKU are at risk to develop neurological complications [14]. Although neurological signs and symptoms are generally more prevalent and severe in untreated and late-treated adult patients [15–21], it is estimated that 20–40% of early treated patients with PKU still suffer from mild neurological complications [15, 22–26]. The most commonly reported neurological signs and symptoms in early treated AwPKU are ataxia, tremor, clumsiness, epilepsy, brisk tendon reflexes, spastic paraparesis and visual impairment, but the presentation and severity is heterogeneous among patients [23, 25, 27–40]. Out of these signs and symptoms, tremor has been best characterised in early treated AwPKU, presenting predominantly as postural and/or kinetic distal tremor of the upper limbs, with some evidence pointing to a deficient dopaminergic system as the underlying origin [19, 24]. In addition to varying severity, the prevalence of neurological signs and symptoms differs between the identified studies, with most studies including a mixed population of early treated AwPKU either on diet, off diet or following a relaxed diet, not allowing comparison between treatment groups. Koch et al. (2002) was the only study to categorise early treated AwPKU depending on their treatment history, showing that neurological signs and symptoms (primarily muscle tone and deep tendon reflex changes) were present in 23% of patients who discontinued diet, while being absent in those who did not relax the diet [30]. In contrast to these findings, other studies did report the presence of tremor and brisk tendon reflexes in AwPKU who were continuously treated, albeit blood Phe concentrations in these patients were generally above the European recommended target range (i.e. > 600 µmol/L) [23, 33, 35, 41]. The experts suspect that even well-controlled AwPKU can develop mild neurological signs and symptoms. However, due to the absence of studies including a cohort of adult patients with metabolic control, there is no evidence yet to support this opinion.

Although historical and concurrent blood Phe concentrations were found to correlate with white matter abnormalities as assessed by magnetic resonance imaging (MRI), a relationship between blood Phe concentrations with the severity of neurological deficits was not consistently identified by the included studies; neither were neurological signs and symptoms correlated with the extent of MRI abnormalities [15, 17–19, 24, 29, 38, 42, 43]. The reason for this discrepancy remains unclear but it has been hypothesised that brain Phe concentrations are a more reliable predictor of neurological outcomes, supported by the fact that interindividual differences have been reported in the uptake kinetics of blood Phe at the blood–brain barrier [41, 44, 45]. However, other mechanisms have been postulated to explain the lack of correlation between blood Phe concentrations with neurological complications, including the blood Phe-independent increased availability of the striatal D2/3 receptor, presumably due to dopamine depletion [46]. In addition, the vulnerability of certain brain regions to the neurotoxic effects of Phe might change during ageing. This has been demonstrated in late-diagnosed cases of PKU in whom neurological signs and symptoms did not emerge before adulthood, despite being untreated throughout childhood and adolescence [47].

#### Onset of neurological signs and symptoms

**Table:** 

**In some cases, neurological signs and symptoms can develop slowly over several years upon treatment discontinuation, while in other cases neurological deficits can occur acutely after stopping treatment. Regardless of the time to onset, the presentation of neurological signs and symptoms is heterogeneous**	

Early diagnosed and treated patients frequently start to relax the Phe-restricted diet between childhood and adolescence, putting them at risk to develop neurological complications. The time frame during which symptoms can emerge largely varies between weeks up to 20 years after treatment discontinuation [15, 17, 23, 25, 28, 34, 36, 38, 41, 42, 48–50]. As a result, neurological signs and symptoms often do not become apparent before adulthood, albeit no correlation between the length of treatment discontinuation and neurophysiological markers was found [23]. Tremor appears to be one exception to this rule. A large cross-sectional study noted that, in PKU, tremor onset is frequently before the age of 10 years (mean age at onset: 13.7 ± 8.1 years) [24]. It was further demonstrated that tremor severity did not correlate with blood Phe concentrations, though the study did not compare the level of metabolic control in patients with tremor versus those without. This comparison was made by another study, showing that the index of dietary control (i.e. mean of all half-year medians of Phe) during the first 6 years of life was slightly out of range (> 360 µmol/L) for patients both with and without tremor, confirming that other pathogenic mechanisms may be at play [19].

Late-diagnosed patients or patients with poor metabolic control during infancy usually present with early onset developmental delay, reduced intelligence and/or seizures that trigger diagnosis of PKU not necessarily coinciding with the presence of any other neurological symptoms or signs [1, 36]. If these patients are diagnosed and treated in childhood, neurological deficits, such as tremor, ataxia, seizures, paraparesis, stereotypies and tics, can still occur in adulthood after a period of treatment discontinuation [1, 25, 34, 36, 47, 50, 51]. In some cases, these late-onset complications can be severely disabling and progressively worsening, not only in late-diagnosed but also in early treated AwPKU upon early treatment discontinuation in childhood or adolescence [25, 34, 36].

#### Reversibility of neurological signs and symptoms

**Table:** 

**In PKU, neurological signs and symptoms (e.g. tremor, brisk tendon reflexes and/or seizures) that develop in adulthood are potentially reversible upon achieving or regaining metabolic control, depending on how long they have been present**	

Although evidence on the relationship between blood Phe concentrations and the severity of neurological manifestations is conflicting, resumption of the Phe-restricted diet upon discontinuation was consistently shown to improve neurological signs and symptoms (e.g. tremor, seizures and brisk tendon reflexes) in early treated patients [15, 25, 34, 48, 50, 52, 53]. While in some patients symptoms can be fully reversed, others show at least partial improvement in most severe and acute neurological complications [25, 34]. Furthermore, the reversibility of symptoms is not limited to neurological manifestations. A recent study by Burgess et al. (2021) showed that even after a mean time off dietary control of 19.1 years, both cognitive and psychiatric (anxiety, depression) outcomes were attenuated in early treated AwPKU after resumption of the Phe-restricted diet for 12 months [54]. Similar findings have been reported for pharmacological treatments, sapropterin dihydrochloride and pegvaliase, improving symptoms of inattention and deficits in executive function in early treated patients with PKU [55, 56]. The underlying mechanisms remain to be elucidated but it is hypothesised that restoration of monoaminergic signalling and remyelination contribute to the improvement of PKU-associated neurological manifestations [54, 57]. This has been confirmed in studies showing amelioration of microstructural white matter integrity and neural activity upon reduction of blood Phe [58–60].

Even in patients with a late diagnosis and, hence, partially irreversible damage, clinical improvement in neurological signs and symptoms was noted upon initiation of the Phe-restricted diet during childhood or resumption of diet after a period of discontinuation [1, 25, 53, 61]. However, signs and symptoms that already emerged in childhood or adolescence can only be improved to some extent upon the re-introduction of the Phe-restricted diet in adulthood, while complete amelioration of neurological signs and symptoms is mostly limited to those that have developed late, emphasising the importance of both early and continuous treatment.

### Differential diagnosis of neurological signs and symptoms in PKU

**Table:** 

**Early treated adults with PKU who have been lost to follow-up may present to a neurologist without recognising PKU as the underlying cause of the neurological signs and symptoms**	

Early treated AwPKU who have been lost to follow-up are no longer in contact with their metabolic physician and often have blood Phe concentrations well above the target ranges recommended by the guidelines [62]. As a result, they are at risk for late-onset neurological signs and symptoms but may, however, not be aware of the association of these complications to PKU and consult a neurologist instead of a metabolic physician when presenting with neurological deficits. In addition, patients may not proactively mention their diagnosis of PKU due to the historical recommendation to stop treatment at 12–18 years of age, cognitive impairment and/or displacement because of childhood traumatic experiences, among other reasons [62, 63]. The differential diagnosis is further complicated by neurologists often not being aware of PKU being a potential cause of neurological signs and symptoms in adulthood. Therefore, a clear image of a patient’s medical history is crucial to identify the disease underlying the neurological complications, and should include questions that focus on childhood, dietary habits and intellectual development. If patients present with more severe and progressive neurological signs and symptoms, substantial developmental/intellectual delay and psychiatric comorbidities, late diagnosis of PKU should be considered. However, late diagnosis is becoming limited to patients living in countries without newborn screening programmes for PKU (e.g. Albania, Morocco, Hong Kong, India) or patients diagnosed after their implementation, which in most, albeit not all, European countries only applies to older adults above the age of 50 (e.g. Austria, Germany, France, Spain, United Kingdom) [64–66]. In addition, false negative results should be considered, though these are rare [67].

Due to the heterogeneous presentation of neurological symptoms, no single symptom can be identified as a red flag for neurologists to consider PKU in the differential diagnosis. Instead, the combination of usually mild neurological signs and symptoms (e.g. ataxia, hyperreflexia, tremor) together with deficits in cognitive functioning (e.g. cognitive decline, impaired executive functions) and/or psychiatric symptoms (e.g. depression, mood swings, anxiety disorders, obsessive–compulsive disorders) should trigger neurologists to test for PKU. Depending on the symptoms, neurological examination should focus on movement disorders and/or assessment of psychiatric symptoms. The Montreal Cognitive Assessment (MoCA) is recommended by the expert panel to confirm the presence of deficits in cognitive functioning [68]. If the MoCA is abnormal, a full cognitive assessment should be performed by a neuropsychologist along with an MRI and lumbar puncture if needed for the differential diagnosis. Regarding MRI, bilaterally decreased mean diffusivity in the white matter tract is indicative of PKU, with largest changes most commonly occurring in patients who have discontinued treatment for several years, correlating both with age and the level of metabolic control [69–71]. Nevertheless, the extent of white matter abnormalities does not seem to explain the severity of cognitive or neurological deficits, further complicating the differential diagnosis with multiple metabolism-related leukoencephalopathies that should be considered [72]. Hence, elevated blood Phe concentrations and genotyping provide the only confirmation of PKU. Additional assessments that can aid the differential diagnosis of PKU are the measurement of motor, sensory and visually evoked potentials. Although latencies are not always predictive of neurological signs and symptoms, they do seem to correlate with the level of metabolic control [23, 73]. Lastly, an electroencephalogram should be performed in patients with suspected seizures or a history of seizures [51].

An overview of these findings has been included in a leaflet (Supplementary Information), summarising the neurological symptoms typically experienced by early treated AwPKU and providing a list of recommended examinations. The leaflet emphasises the treatability of PKU and the possible reversibility of symptoms. The leaflet will be disseminated via neurology conferences and the organisation of educational and training events, using video case examples. The focus will be, first, to raise awareness among neurologists in Germany and Austria, but the leaflet can later be disseminated in other countries. Besides dissemination of the leaflet, dried blood spot testing cards will be distributed at neurological practices to provide easy access to diagnostics and further increase awareness about PKU.

### Multidisciplinary care in PKU

**Table:** 

**To better understand the neurological and psychiatric signs and symptoms experienced by adults with PKU and optimally adjust patients to treatment, it is highly recommended to include neurologists in the PKU multidisciplinary care team, ensuring regular consultation between metabolic physicians, including paediatricians due to the lack of adult centres, and the neurological team**	

The expert panel (highly) recommends including a neurologist in the multidisciplinary team, especially when neurological deficits are suspected by the metabolic team. Although approximately half of all adult patients with PKU remain to be seen by metabolic experts, many do not achieve sustained metabolic control, despite active follow-up [5, 6]. Therefore, these patients can still experience various PKU-associated manifestations, including neurological ones, and should be referred by the metabolic physician for neurological examination. Depending on the symptoms, an orientating neurological exam can be performed first by the metabolic physician in a few minutes to determine if follow-up by the neurological team is needed. Preliminary assessments that can be performed by the metabolic team include: Archimedes spiral and tremor scales (e.g. Fahn-Tolosa-Marin Tremor Rating Scale [74]), ataxia scales (e.g. Scale for Assessment of and Rating of Ataxia [75]), MoCA [68], index finger tapping rate, Movement Disorder Society-Sponsored Revision of the Unified Parkinson’s Disease Rating Scale [76], Frontal Assessment Battery [77] and Beck Depression Inventory [78]. Although deficits may be flagged by these initial screening assessments, close cooperation between metabolic physicians and neurologists should be improved, as it is currently limited or even completely lacking in many centres. This can lead to under-reporting of neurological symptoms, not accurately reflecting the full spectrum of complications experienced by AwPKU with many studies including only small patient cohorts. Furthermore, AwPKU are often still treated in paediatric clinics with limited knowledge about neurological diseases, contributing to the under-recognition and/or under-treatment of neurological signs and symptoms. A registry run by centres for rare diseases would be very useful to capture the presence of neurological signs and symptoms in early treated AwPKU, especially if neurologists are included in the treatment team. In addition to neurologists, PKU care should ideally involve different specialities and professions, such as dietitians, neuropsychologists, psychiatrists, endocrinologists, neuroradiologists, occupational therapists, physiotherapists and speech therapists when there is a clinical need.

The benefits of including neurologists in the multidisciplinary team are twofold. As mentioned, metabolic physicians may lack experience to recognise neurological complications and may not apply the proposed neurological assessments. Including a neurologist in the multidisciplinary team will help with the diagnosis and treatment of neurological signs and symptoms present in AwPKU. Neurologists can detect early signs of neurological deterioration and thus prevent outcomes of suboptimal and overtreatment, also considering micronutrient deficiencies. Decreased vitamin B12 levels are not uncommon among patients with PKU, albeit generally less important than the degree of metabolic control [39, 40]. Regarding treatment, current blood Phe target values are somewhat controversial due to the lack of evidence in studies that include early treated AwPKU with metabolic control. In addition, some patients are more resilient to the detrimental effects of brain Phe and hence, overtreatment in these patients should be avoided. Nevertheless, it should be considered that neurological deficits sometimes only occur several years after being exposed to elevated Phe concentrations, arguing for lifelong metabolic control [2, 4, 47]. On the other hand, neurologists recognising PKU as the underlying disease in symptomatic neurological patients can help to transfer AwPKU, who have been either lost to follow-up or late-diagnosed, to specialised metabolic centres with the aim of regaining/achieving metabolic control to ameliorate or even fully reverse the neurological signs and symptoms that emerged in adulthood. Frequent blood Phe monitoring, discussion of treatment options and regular neurological, cognitive and/or psychiatric assessments are the hallmarks of follow-up of which the frequency should be decided in a joint effort between the neurologist and metabolic physician.

### Patient cases

An overview of three adult patient cases is provided in Table 1. All patients were diagnosed with PKU at birth and treated immediately thereafter. However, after treatment was discontinued in adolescence, neurological deficits emerged for which they presented themselves at the neurological clinic after being lost to follow-up.

**Table 1:** Characteristics of three adult patient cases with early treated PKU

Patient	Sex-age (year)	Age at diet discontinuation (year)	Phe at presentation of neurological symptoms (µmol/L)	Leukoencephalopathy (MRI)	Improvement of neurological symptoms upon diet resumption	Phe at 6 months after diet resumption (µmol/L)
Patient 1	F-50	14	1816	+	+	726–969
Patient 2	M-41	17	1271	+	+	1029
Patient 3	F-37	12	1210	NP	+	≤ 605

+ :confirmed, *NP* not performed

#### Patient 1

**Fig. 2:**
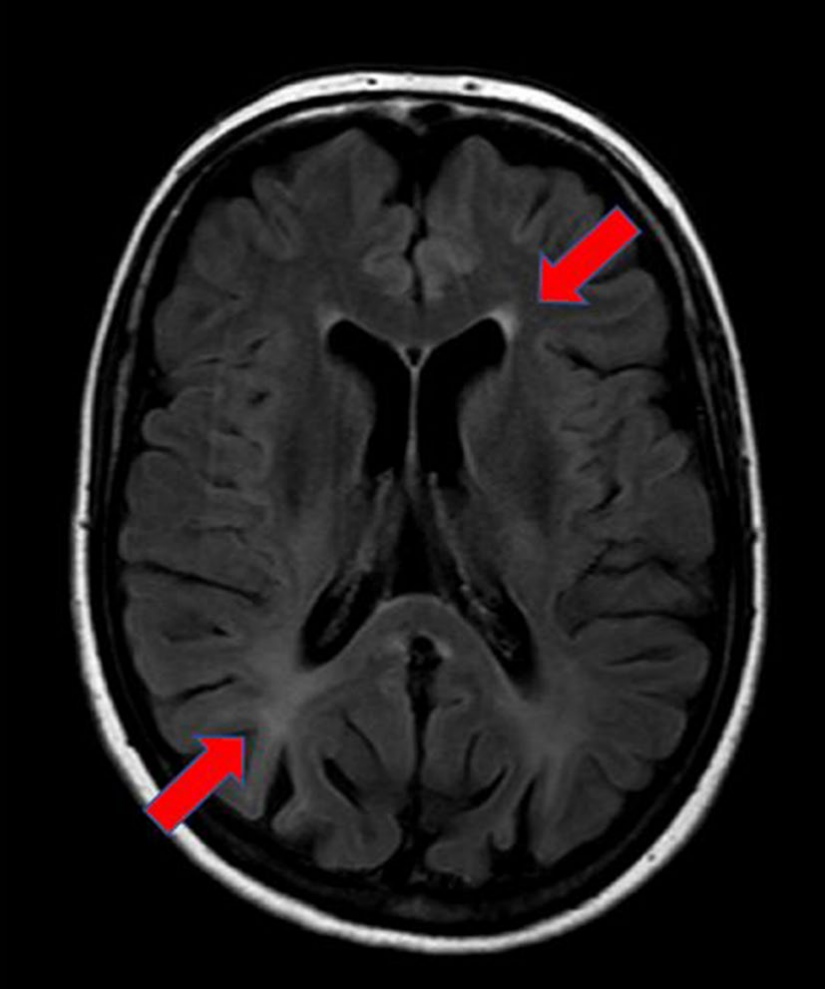
MRI (FLAIR) of a 50-year-old patient with PKU who was early diagnosed and treated but discontinued diet at the age of 14. Arrows point to periventricular white matter hyperintensities, a sign of metabolic (e.g. PKU-related) encephalopathy

The female patient in her 50s (age: 50 years; weight: 54 kg; height: 174 cm) was early diagnosed with PKU (no genetic information available) and strictly treated until the age of 14 after which the diet was relaxed and no supplements were taken. The patient was consuming a normal diet without protein restriction. The patient presented herself at the neurological clinic with dysarthria, pyramidal signs, intention tremor, pathological leg-extension test and spastic-ataxic gait pattern. In addition, finger–nose test and heel–shin test revealed marked ataxia. With regard to the technical findings, sensory evoked potentials could not be evoked, whereas electroneurography showed no signs of polyneuropathy and MRI of the spinal cord was normal. However, periventricular white matter lesions were detected by cranial MRI (fluid-attenuated inversion recovery; FLAIR) (Fig. 2). Based on the combination of these symptoms and examination, the patient was diagnosed first with metabolic encephalopathy of unknown aetiology, clinically encompassing ataxia and spastic paraparesis.

Three months later, the patient presented with cachexia, spastic paraparesis and maculopapular rash. The patient was not able to walk independently and sat in a wheelchair. Laboratory blood test results showed elevated concentrations of ornithine (204 µmol/L; 2.7 mg/dL), Phe (1816 µmol/L; 30 mg/dL) and a nutritional deficiency (vitamin D and vitamin B12). Following the measurement of elevated blood Phe, the patient was diagnosed with PKU-related metabolic encephalopathy and dietary management was re-introduced. Six months after resumption of the Phe-restricted with dietary supplements, blood Phe concentrations decreased to 726–969 µmol/L (12–16 mg/dL). In addition, neurological deficits and skin lesions completely resolved without the need for using walking aids and the ability to restart working in a nursing home.

#### Patient 2

Male patient in his forties (age: 41 years; weight: 85 kg; height: 183 cm) was early diagnosed with classical PKU (*PAH* gene: c.[878 T > A];[1222C > T], p.[Lys293*];[Arg408Trp]). The variant 1222C > T was previously described as causing severe classical PKU without responsiveness to tetrahydrobiopterin and less than 1% residual PAH activity. In addition, the variant 878 T > A results in a stop codon. The patient followed a strict diet until the age of 10 after which the diet was relaxed and completely discontinued at the age of 17. The patient had a secondary school diploma. At presentation, the patient was consuming a flexible vegetarian diet with a daily protein intake of 60–80 g without consuming any protein supplements. Blood Phe concentration at presentation was 1271 µmol/L (21 mg/dL). The patient presented with neurological deterioration with bilateral progressive visual loss, progressive motor disturbances and tetraspasticity. The patient required a wheelchair, as standing without aid was impossible. The patient was a driver but had to quit his job because of severe visual impairment and paraplegia. The T2-weighted turbo inversion recovery magnitude (TIRM) sequence showed confluent hyperintensities extending from the periventricular white matter to the occipital cortex, which was slightly progressive in comparison to a previous recording. In addition, the apparent diffusion coefficient (ADC) was reduced in the edge area of the lesions, corresponding to the progressive parts. Brainstem and cerebellum were normal. After resumption of the Phe-restricted diet with an intake of 10–15 g of protein/day that was supplemented with 60 g glycomacropeptide, symptoms almost immediately improved. Six months later, the patient’s blood Phe concentration remained high but was reduced to 1029 µmol/L (17 mg/dL). He regained most of his eyesight, was able to walk and drive but fine motor skills remained impaired. As blood Phe remained high, the patient initiated treatment with pegvaliase.

#### Patient 3

Female patient in her thirties (age: 37 years; weight: 57 kg; height: 159 cm) who was diagnosed with PKU via newborn screening with a genotype (c.[754C > T];[1162G > A], p.[Arg252Trp];[Val388Met]) known to cause mild-to-severe and severe PKU. During her first days of life, she experienced a cardiac arrest (most likely unrelated to the PKU), resulting in minor mental disability. Strict dietary treatment was implemented immediately after diagnosis but discontinued at the age of 12 when the patient was living in a foster home. At initial presentation, the patient was consuming 40–50 g of protein/day resulting in blood Phe ranges of 908–1210 µmol/L (15–20 mg/dL). She resided in an assisted living group and was employed in a workshop for disabled individuals. Regarding clinical findings, the patient was suffering from severe depression and her communication skills were severely compromised. Lab results showed deficiencies in iron and essential amino acids without detectable vitamin D or vitamin B12 deficiency. With the help of her care takers, a more restricted diet with Phe-free protein supplements was reintroduced, resulting in blood Phe concentrations ≤ 605 µmol/L (≤ 10 mg/dL). Six months later, the patient was consuming 20–30 g of protein/day and 42 g of Phe-free protein supplements. Over the next 3 years, the patient’s overall and mental health improved substantially. At a subsequent visit, she was alert, without clinical depression, and fully taking part in the conversation. In addition, she was offered a new job with more responsibility. At the last visit, she was able to manage the Phe-restricted diet by herself, while having awareness that, with higher blood Phe, she suffers from nervousness, restlessness, and can “barely think” (cited by patient). In addition, she plans to move into an apartment of her own and start living independently.

## Discussion

This systematic review of the literature emphasises that neurological abnormalities are not limited to late-diagnosed patients with PKU. If treatment is discontinued after childhood or if the diet is not sufficient to maintain blood Phe concentrations within range, multiple neurological signs and symptoms can emerge in adulthood [23, 25, 27–40]. Although these signs and symptoms are usually mild, they can progressively worsen as illustrated by the herein presented patient cases. Based on the literature review and patient cases, neurological complications in early treated AwPKU are often related to the motor system such as ataxia, tremor and spastic paraparesis, but visual impairment, seizures, deficits in cognitive functioning and psychiatric manifestations have been reported as well. While the exact mechanism is still unknown, most studies associate the emergence of neurological manifestations to the neuropathophysiology of PKU that is characterised by reduced monoaminergic neurotransmitters (serotonin, dopamine, noradrenaline) in the brain and abnormal cerebral myelination, apparent as white matter lesions [3]. Besides the fact that high blood Phe concentrations disturb the uptake of amino acids at the blood–brain barrier, impaired myelination negatively affects synaptic dopamine concentrations [3]. In addition, the activity of tyrosine hydroxylase, the key regulatory enzyme of dopamine synthesis, was found to be reduced in non-clinical studies [79]. However, it is surprising that white matter abnormalities, though correlated with blood Phe concentrations, do not predict the severity of neurological deficits, and hence, there appears to exist an unexplained variability between individuals to the neurotoxic effects of high Phe concentrations [15, 17–19, 24, 29, 38, 42, 43]. Interindividual differences in the blood-to-brain Phe ratio may partially explain this variability [41], but anecdotal cases of patients with high brain Phe and favourable outcomes have been reported as well [47]. There also seems to be an age-dependent factor with long-term exposure to high blood Phe concentrations being predictive not only of white matter but also of grey matter changes, albeit studies remain limited to the paediatric and young adult PKU population [80, 81]. Despite the neuropathophysiological mechanisms needing further research to explain this variability, evidence consistently shows that brain changes and neurological deficits are at least to some extent reversible upon lowering of blood Phe [15, 25, 34, 48, 50, 52, 53]. However, the most optimal blood Phe target in adulthood remains a matter of debate, with current European and American PKU treatment guidelines differing in their recommendations (European target: 120–600 µmol/L; US target: 120–360 µmol/L) [2, 4]. Although the field would benefit from an international effort harmonising this treatment target, there is currently insufficient evidence to conclude which levels would be safe in adulthood, especially because, to date, nearly all studies include an adult PKU population with average blood Phe concentrations above the European target. Pharmacological treatments that allow patients to achieve lower or even physiological blood Phe concentrations may change the treatment paradigm if a substantial benefit can be shown, but until these become widely adopted, the majority of patients will continue to rely on consuming a Phe-restricted diet to maintain blood Phe concentrations at least below 600 µmol/L in the long term.

Due to the prevalence of neurological complications, it is crucial that neurologists are familiar with the heterogeneous presentation of signs and symptoms experienced by AwPKU. Recent publications have made similar attempts to raise awareness of PKU and its neurological and/or neuropsychiatric complications among the neurological community [1, 25]. This is especially important for patients who have become lost to follow-up and are possibly undertreated. Neurologists can be the first point of contact for lost to follow-up patients who experience late-onset neurological complications, as these patients may not associate these signs and symptoms to their earlier diagnosis of PKU. In these patients, the presence of motor signs and symptoms combined with cognitive impairment and/or psychiatric symptoms should be considered red flags for PKU, of which a diagnosis can only be confirmed by measurement of blood Phe concentrations followed by genotyping. MRI may further reveal white matter abnormalities but these can be similar to other leukodystrophies [72]. After confirming the diagnosis, the neurologist should refer the patient to the metabolic team with the aim of resuming treatment for PKU. If needed, the neurologist can remain involved in the follow-up and perform regular neurological examinations to evaluate the disease progression. Regarding examinations, it should be noted that there is a lack of validated screening tools for the adult population. In addition, AwPKU who have been exposed to high blood Phe often experience impairment in neurocognition that can further complicate the completion of self-assessments [82]. Therefore, it is recommended to combine these assessments with more objective performance-based tasks, such as the index finger tapping rate and Archimedes spiral [83]. Advanced imaging can also be considered to understand the disease progression, though the severity of neurological signs and symptoms do not always correlate with the extent of white matter abnormalities [15, 17–19, 24, 29, 38, 42, 43]. For patients with neurological manifestations who remain in follow-up, it is furthermore recommended to include neurologists in the multidisciplinary team, as they can support less-experienced metabolic providers, including paediatricians, with the identification and management of these complications [84]. Regardless of whether patients are late diagnosed or early treated, management should focus on regaining or achieving metabolic control, as signs and symptoms are often partially or even fully reversible [1, 15, 25, 34, 48, 50, 52, 53, 61].

Because evidence is mostly based on case reports, neurological complications in PKU may currently remain unrecognised and under-reported [1, 25]. The field would benefit from registry studies, including both metabolic and neurological practices, which may change the findings of the herein presented SLR. In addition, the expert panel consisted of German and Austrian physicians and hence, recommendations may not be applicable to other countries or regions, especially those with better adult services for patients with PKU.

The findings of the systematic review and opinions of the expert panel have been summarised in a leaflet that will be disseminated together with dried blood spot cards among the neurological community first within Germany and Austria and later in other countries. Phe levels, however, can also easily be determined in regular laboratories without the need to submit dried blood spot cards. These efforts are intended to shorten the diagnostic delay of late-diagnosed patients and, more importantly, reduce the high number of lost to follow-up patients. Because of the prevalence of neurological symptoms in AwPKU, neurologists should be an integral part of the PKU treatment team, understanding that progressive worsening of symptoms can be prevented through re-establishing blood Phe control as soon as symptoms emerge.

## Supplementary Information

Below is the link to the electronic supplementary material.

Supplementary file1 (DOCX 51 KB)

Supplementary file2 (PDF 27 KB)

## Data Availability

The data that support the findings of this study are available from the corresponding author upon reasonable request.

## Abbreviations

ADC: Apparent diffusion coefficient
AwPKU: Adults with phenylketonuria
FLAIR: Fluid-attenuated inversion recovery
LAT: L-Type amino acid carrier
MoCA: Montreal Cognitive Assessment
MRI: Magnetic resonance imaging
PAH: Phenylalanine hydroxylase
Phe: Phenylalanine
PICOS: Population, Intervention, comparator, outcomes, study types
PKU: Phenylketonuria
PRISMA: Preferred Reporting Items for Systematic Reviews and Meta-Analysis
RCT: Randomised controlled trial
SLR: Systematic literature review
TIRM: Turbo inversion recovery magnitude
