# Supplementary material for: Characterisation and differential diagnosis of neurological complications in adults with phenylketonuria: literature review and expert opinion
Source: J Neurol. 2023 Apr 20;270(8):3675–87. doi: 10.1007/s00415-023-11703-4 (PMC10345006; doi:10.1007/s00415-023-11703-4)
Supplement: Supplementary file 1 — Supplementary file1 (DOCX 51 KB) [file 415_2023_11703_MOESM1_ESM.docx]

**Overview of cross-sectional studies.**

| **First author (year)** | **Sample** | **Diagnosis/Treatment initiated/discontinued** | **Frequency of neurological symptoms** | **Metabolic control/ Blood Phe** | **Relation to metabolic measures** |
| --- | --- | --- | --- | --- | --- |
| Cleary  (1994) | **77 PKU patients**  Aged between 14-49 yrs | **Diet initiation:**  70 patients started diet before 2 months of age  5 patients were late-diagnosed  2 patients diagnosed at antenatal screening  **Diet discontinuation:**  58 patient discontinued diet around the age of 14 years | **Only evaluated in patients who discontinued diet:**  - Brisk tendon reflexes 10% (6/58)  - Tremor and brisk tendon reflexes 7% (5/58) | **-** | - |
| Dezortová  (2001) | **15 ET AwPKU**  Mean age: 23.4 (4.6) yrs  **14 HC**  Mean age: 21.5 (5.9) yrs | Not specified | **ET AwPKU**  Tremor: 20% (3/15)  **HC**  No tremor | **ET AwPKU**  Mean blood Phe:  1503.8 (309.7) µmol/L  **HC**  Mean blood Phe:  117.9 (57.9) µmol/L | - |
| Hvas  (2006) | **31 AwPKU**  Median age: 28 yrs (range: 18-43) | Less-protein restricted diet with (N=24) or without (N=7) supplementation of PreKUnil | **Neurological symptoms in 43% (13/30)**  Paraesthesia most common neurological symptom: 30% (9/30) | - | Patients with at least one neurological symptom tended to have lower serum cobalamins. The presence of symptoms was not associated with other biochemical markers determined |
| Nardecchia (2019) | **59 ET PKU patients**  Mean age: 23.2 (9.8) yrs  **43 HC**  Mean age: 23.1 (12.0) yrs | Diet | **ET PKU patients**  Tremor: 32% (19/59)  **HC**  No symptoms | - | No correlation between measures of quality of metabolic control and tremor severity score  Correlation between prolactin and tremor severity and measures of metabolic control |
| Pfaendner  (2005) | **31 ET AwPKU**  Mean age men  (N=17): 30 yrs  Mean age women (N=14): 28 yrs  **27 HC**  Mean age men  (N=15): 30 yrs  Mean age women (N=12): 27 yrs | Off diet: 11/31 (35%)  Relaxed diet: 10/31 (32%)  Strict diet: 10/31 (32%)  Age at diet relaxation: ≥18 years | **ET AwPKU**  Tremor: 42% (13/31)  **HC**  Not specified | **Mean blood Phe**  Current:  1276.5 (490.0) µmol/L  Until 5 years:  308.6 (102.2) µmol/L  Until 12 years:  399.3 (163.3) µmol/L | - |
| Pietz  (1998) | **57 ET AwPKU**  Mean age: 23.6 yrs (range: 17-33)  **48 HC**  Mean age: not specified (range: 16-35) | Diet onset at mean age of 34.2 days (≤3 months) | **ET PKU patients**  - Tremor: 28% (16/57) - Brisk tendon reflexes:  2% (1/57)  **HC**  Tremor: 15% (7/48) | **Mean blood Phe**  Current:  1085 (303) µmol/L  Until adult:  676 (157) µmol/L  Until 12 years:  424 (158) µmol/L  From 12 years until adult: 964 (194) µmol/L | No difference between patients with and without tremor with regard to the onset of dietary treatment, differences in indices of biochemical control or concurrent blood Phe concentrations |
| Robinson  (2000) | **83 ET PKU patients**  Median age:  22 yrs (off diet);  21 yrs (relaxed);  24 yrs (strict)  (range: 11-38) | Off diet: 31/83 (37%)  Relaxed diet: 30/83 (36%)  Strict diet: 22/83 (26%) | Tremor: 12% (10/83)  Brisk tendon reflexes: 41% (34/83) | **Mean blood Phe**  Off diet:  1200 µmol/L  Relaxed diet:  1100 µmol/L  Strict diet:  500 µmol/L | No relationship between vitamin  B12 and the presence or absence of  brisk reflexes or tremor |
| Thompson  (1993) | **25 ET PKU patients**  **9 LT PKU patients**  Aged between 8-33 yrs | 16 ET patients on diet  2 LT patients on diet  Others discontinued diet between 7-18 years (ET patients) and 8-20 years (LT patients) | **ET PKU patients (symptoms in 10 subjects)**  - Tremor: 24% (6/25)  - Brisk tendon reflexes:  20% (5/25)  - Epilepsy: 8% (2/25)  **LT PKU patients (symptoms in all subjects)**  -Tremor: 56% (5/9)  - Hyperreflexia:  100% (9/9)  - Epilepsy: 22% (2/9) | - | ET PKU patients: No relationship between MRI grade, neurological findings, mean blood Phe levels during the first 8 years of life or time lapsed since stopping the low Phe diet but correlation between MRI grade and concurrent blood Phe concentrations |

**Overview of cohort studies.**

| **First author  (year)** | **Study design** | **Sample** | **Diagnosis/Treatment initiated/discontinued** | **Frequency of neurological symptoms** | **Metabolic control/ Blood Phe** | **Relation to metabolic measures** |
| --- | --- | --- | --- | --- | --- | --- |
| Bilder (2017) | Retrospective | **3714 AwPKU  (ET and LT)**  Mean age: 38.5 yrs  **7060 DM**  Mean age: 38.1 yrs  **22726 GP**  Mean age: 41.3 yrs | Not specified | **Whole cohort**  Movement disorders/ Parkinson’/tremor:  7.7% PKU – 6.7% DM – 3.3% GP  Epilepsy & convulsions; 5.2% PKU - 4.8% DM - 2.3% GP  **Age range 20-39**  Movement disorders/ Parkinson's/tremors; 3.3% PKU - 3.7% DM - 1.4% GP  Epilepsy & convulsions; 2.4% PKU - 4.7% DM - 1.5% GP | **-** | - |

| González  (2011) | Retrospective | **121 PKU patients**  Median age: 16 yrs (range 1 month –  46 yrs)  **92 ET PKU patients**  Median age: 11 yrs (range 1 month –  39 yrs)  **29 LT PKU patients**  Median age: 34 yrs (range 15- 46 yrs)  47% adults (54/121) | Diet: 88% (107/121) Sapropterin: 12% (14/121)  All patients were treated with Phe-restricted diet at diagnosis. Fifteen patients abandoned the diet after the age of 6–10 years and all of them resumed diet with 7 of them being early diagnosed | **ET patients (N=92)**  - Epilepsy: 1% (1/92)  - Tremor: 12% (11/92)  - Clumsiness: 11% (10/92)  **LT patients (N=29)**  - Epilepsy: 31% (9/29)  - Tremor: 93% (27/29)  - Clumsiness: 89% (26/29)  - Spasticity: 28% (8/29) | **ET – median IDC**  Until 6 years: 310 (105-992) µmol/L  Past year: 348 (104-1127) µmol/L  **LT – median IDC**  Past year: 433 (260-1247) µmol/L | The proportion of patients with neurological complications was significantly different in patients with good, intermediate and poor IDC in the first 6 years of life and those in the immediately past year |
| --- | --- | --- | --- | --- | --- | --- |
| Koch  (2002) | Prospective | **73 ET AwPKU**  ≥21 years of age | Discontinued diet:  88% (64/73)  On diet:  12% (9/73) | **Discontinued diet**  - Convulsions: 2% (1/59)  - Muscle tone and deep tendon reflex changes: 24% (12/51)  **On diet**  No symptoms | **Age at diet discontinuation 4.8-6.5 years:**  Current: 1448 (465)  **Age at diet discontinuation 6.5-12.5 years:**  Current: 1510 (602)  **Age at diet discontinuation 12.5-20.0 years:**  Current: 1268 (470)  **No discontinuation**  Current: 926 (268) | - |
| Pedersen  (1974) | Prospective | **58 PKU patients**  Mean age: 27 yrs (range: 4-69) | None of the patients in the longitudinal study received diet during the observation period, but 2 patients on diet for shorter period of time and 4 patients received diet at the time of examination | **Neurological symptoms in 79% (46/58) patients**  - Brisk tendon reflexes: 38% (22/58)  - Hypotonia: 10% (6/58)  - Parapalgia: 3% (2/58)  - Hemiplegia: 2% (1/58)  - Epilepsy: 33% (19/58)  - Hyperkinesia: 3% (2/58)  - Optic atrophy: 11% (5/58)  - Strabismus: 14% (8/58) | **-** | - |
| Schuett  (1985) | Retrospective | **72 PKU patients**  Age range not specified | Nearly all patients on diet since infancy  All patients returned to diet after discontinuation  Median age for initial diet discontinuation:  6-6.5 years (range: 3-20) | Improvement in tremor and seizures upon diet resumption | **-** | No significant correlation between number of observed problems off diet and either age of patient at time of diet discontinuation or length of discontinuation  Significant correlation between improvement on electroencephalogram with length of diet resumption |
| Yannicelli  (1995) | Retrospective | **88 AwPKU**  Age range not specified | 59% started diet after the age of 35 | Improvement in incidence of neurological symptoms in 26% and seizures in 11% of patients upon diet resumption | Blood Phe reduction from 1659 (1000-2330) µmol/L to 617 (182-1495) µmol/L upon diet resumption | - |

**Overview of case-control studies.**

| **First author  (year)** | **Sample** | **Diagnosis/Treatment initiated/discontinued** | **Frequency of neurological symptoms** | **Metabolic control/ Blood Phe** |
| --- | --- | --- | --- | --- |
| Pérez-Dueñas  (2006) | **27 PKU patients**  Mean age: 20 (7) yrs (range: 9-37 yrs)  16 ET PKU patients (range: 9-31 yrs)  11 LT PKU patients (range : 19-31)  **27 HCs**  Mean age: 21 (7) yrs (range: 10-37 yrs) | 16 ET PKU patients starting diet <2 months of age  11 LT PKU patients starting diet at a mean age of 8 yrs (range: 9 months – 30 yrs) | **ET PKU patients**  - Tremor: 31% (5/16)  - Hyperreflexia: 19% (3/16)  - Clumsiness: 6% (1/16)  **LT PKU patients**  - Tremor: 73% (8/11)  - Hyperreflexia: 27% (3/11)  - Clumsiness: 36% (4/11)  - Hypotonia: 9% (1/11)  - Strabismus: 18% (2/11) | **-** |
| Pietz  (1995) | **8 ET AwPKU**  Mean age: 23 yrs  (range: 21-26)  **13 HCs**  Mean age: 27 yrs  (range: 19-47 yrs) | On diet: 4 patients  Off diet: 4 patients | Tremor in one patient | Baseline blood Phe:  1040 (700-1390) µmol/L |
| Pilotto (2021) | **19 ET AwPKU**  Median age: 41 yrs (range: 35-44)  **25 HC**  Median age: 34 yrs (range: 30-40) | Continuous diet  (+ sapropterin (N=1)):  53% (10/19)  Diet discontinuation for 3-24 years in 9 patients | **ET AwPKU**  - Hyperreflexia: 53% (10/19)  - Kinetic tremor: 21% (4/19)  - Slowed horizontal saccades: 32% (6/19)  - Bradykinesia and hypokinesia: 18% (3/19)  - Supranuclear gaze palsy: 6% (1/19)  - No rigidity  **HC**  No symptoms | **Median blood Phe**  ET AwPKU: 873 (644-1115) µmol/L  HC: 44 (38-50) µmol/L |

| Rupp  (2001) | **17 ET AwPKU**  Mean age: 26 yrs  (range: 18-31 yrs)  **10 HC**  Mean age: 25 yrs  (range: 20-46 yrs) | Off diet: 35% (6/17)  Varying degree of protein restriction in combination with intake of amino acid supplements: 47% (8/17)  Protein restriction without intake of amino acid supplements: 18% (3/17) | Tremor: 18% (3/17) | Mean concurrent blood Phe: 1170 (range: 660-1780) µmol/L |
| --- | --- | --- | --- | --- |

**Overview of case series.**

| **First author (year)** | **Sample** | **Diagnosis/Treatment initiated/discontinued** | **Frequency of neurological symptoms** | **Metabolic control/Blood Phe** |
| --- | --- | --- | --- | --- |
| Burlina  (2000) | **14 ET AwPKU**  Aged between 9-20 yrs | Patients never interrupted diet | Brisk deep tendon reflexes: 21% (3/14)  Brisk deep tendon reflexes and intentional tremor: 14% (2/14)  Brisk deep tendon reflexes, intentional tremor and ankle clonus: 7% (1/14) | Mean blood Phe in patients with abnormal MRI (N=8) ranged between 708-1161 µmol/L |
| Cerone  (1999) | **16 ET PKU patients**  Aged between 14-19 yrs | Off diet at mean age of 11  (6 patients with good metabolic control) | Tremor: 62% (10/16)  Brisk tendon reflexes: 94% (15/16)  Mild ankle clonus: 12% (2/16)  Impaired coordination: 31% (5/16) | Blood Phe concentrations off diet: 1280-3500 µmol/L |
| Crowley  (1990) | **10 AwPKU**  Aged between 18-37 yrs | All patients were off diet but received amino acid supplement for 1 year with dietary adherence being:  - Excellent: 2/10  - Good: 4/10  - Fair: 2/10  - Poor: 2/10 | **Neurological symptoms at baseline in 2 subjects:**  Tremor (N=1) and seizures (N=1) improved after diet resumption | **Mean blood Phe**  Start: 1464 µmol/L  After 3 months: 1410 µmol/L  After 6 months: 1500 µmol/L  After 9 months: 1740 µmol/L |
| Daelman  (2014) | **3 ET AwPKU**  Aged between 26-38 yrs  **2 LT AwPKU**  Aged 40 and 47 yrs | All patients discontinued diet between 5-30 yrs of age (except for one LT PKU case who never initiated diet) | **ET AwPKU**  - Brisk reflexes: 2/3  - Spastic paraparesis: 1/3  - Tremor: 1/3  - Mild ataxia: 1/3  **LT AwPKU**  - Parkinsonism: 1/2  - Tremor: 1/2  - Seizure: 1/2 | Blood Phe >1500 µmol/L when neuropsychiatric symptoms occurred |
| Jaulent  (2020) | **6 ET AwPKU**  Aged between 19-41 yrs  **2 LT AwPKU**  Aged 50 and 54 yrs | **ET AwPKU**  All patients discontinued diet between 7-45 yrs of age  **LT AwPKU**  One patient started diet at age of 2 and discontinued at age of 7  One patient never initiated diet  Diet was resumed in all patients, except one LT AwPKU patients who never initiated diet | **Neurological symptoms present in 8 patients out of 158 AwPKU (5.1%)**  **ET AwPKU**  - Brisk reflexes: 5/6  - Tremor: 4/6  - Ataxia: 4/6  - Dysarthria: 1/6  - Spastic paraparesis: 1/6  - Visual loss: 3/6  - Paraesthesia: 2/6  **LT AwPKU**  - Brisk reflexes: 1/2  - Tremor: 1/2  - Visual loss: 1/2  **Neurological symptoms improved in all patients upon resumption of diet** | **Blood Phe at time of first neurological symptoms or PKU diagnosis if after appearance of first neurological symptoms**  **ET AwPKU**  840-1980 µmol/L  **LT AwPKU**  1200-2640 µmol/L |
| Leuzzi  (1995) | **17 LT PKU patients**  Aged between 3-25 yrs | 12 patients were treated late  (0.7-4.5 yrs) with 4 still on diet at the study examination  5 patients were never treated | **Time of diagnosis**  - Epilepsy: 18% (/17)  **Time of study**  - Tremor: 53% (9/17)  - Pyramidal signs: 23% (4/17)  - Epilepsy: 6% (1/17)  - Hypokinesia: 6% (1/17) | Median blood Phe for year preceding ranged between 343-1820 µmol/L  Median blood Phe at the time of examination ranged between 335-2200 µmol/L |
| Leuzzi  (2000) | **4 ET PKU patients**  Aged between 16-19 yrs  **6 LT PKU patients**  Aged between 15-30 yrs | All patients were off diet at the time of the evaluation  ET patients discontinued diet between 6-19 yrs of age  LT patients discontinued diet between 10-15 yrs of age | **ET PKU patients**  - Tremor: 2/4  - Pyramidal signs: 1/4  **LT PKU patients**  - Tremor: 2/6  - Pyramidal signs: 1/6  - Epilepsy: 1/6  - Nystagmus: 1/6 | **Median blood Phe for year preceding**  **ET PKU patients**  709-1435 µmol/L  **LT PKU patients**  574-1500 µmol/L  **Median blood Phe at the time of examination**  **ET PKU patients**  274-2800 µmol/L  **LT PKU patients**  1039-1820 µmol/L |
| Mainka  (2021) | **26 LT AwPKU**  Median age: 52 yrs (range: 35-57) | Diagnosis (median age:  3 years (range: 1-5.5) was made and/or stable diet was introduced only after 12 weeks after birth  Diet after diagnosis in 88% of patients (23/26)  Diet inconsistent in 54% (14/26) of patients  Diet discontinuation (median duration 18 years) in 35% (9/26) of patients  Median age at diet discontinuation: 14 yrs (range: 5-15) | **Abnormal neurological findings in all patients**  **Movement disorders in 77% (20/26) of patients**  - Ataxia: 54% (14/26)  - Tremor: 38% (10/26)  - Stereotypies: 38% (10/26)  - Tics: 19% (5/26)  - Brisk tendon reflexes: 15% (4/26)  - Hypokinesia: 4% (1/26)  **-** Parkinsonism: 4% (1/26)  **History of seizures in 31% (8/26) of patients** | Median blood Phe at time of last presentation: 720 (306-896) µmol/L |
| Marholin  (19 78) | **6 LT AwPKU**  Aged between 19-53 yrs | All patients resumed diet after a period of discontinuation | Stereotypies in 4/6 patients with improvement in 2 patients upon diet resumption  Tremor in 2/6 patients with improvement in 1 patient upon diet resumption | Resumption of diet lowered blood Phe in 5 patients from 2119-2906 µmol/L to 182-605 µmol/L |
| McDonnell  (1998) | **27 AwPKU**  Mean age 26.1 yrs (range: 18-39 yrs) | **22 ET AwPKU**  Diet initiation between 2-8 weeks of birth  **5 LT AwPKU**  Diet initiation between 8 months to 2 years  All patients but one relaxed diet between the age of 1-22 yrs | **ET AwPKU**  - Tremor: 18% (4/22)  - Hyperreflexia: 54% (12/22)  - Ataxia: 14% (3/22)  - Strabismus: 9% (2/22)  - Optic atrophy: 9% (2/22)  - Extensor planter: 9% (2/22)  - Epilepsy: 4% (1/22)  - Mild dysarthria: 4% (1/22)  - Spastic quadriparesis: 4% (1/22)  - Increased tone leg: 4% (1/22)  **LT AwPKU**  **-** Tremor: 20% (1/5)  - Hyperreflexia: 40% (2/5)  - Increased tone leg: 20% (2/5)  - Optic atrophy: 40% (2/5)  - Ataxia: 40% (2/5)  - Epilepsy: 20% (1/5)  - Divergent squint: 20% (1/5)  - Extensor planter: 20% (1/5) | Median blood Phe for ET AwPKU ranged between 280-2466 µmol/L  Median blood Phe for LT AwPKU ranged between 1146-1654 µmol/L |

| Pérez-Dueñas  (2005) | **54 PKU patients**  Mean age: 19 yrs (range: 6-39 yrs)  36 ET PKU patients  18 LT PKU patients | ET PKU patients started diet within 2 months of age  LT PKU patients started diet at a mean age of 7 yrs and 2 months (range: 3 months-30 yrs) | **Tremor: 41% (22/54; 11 ET and  11 LT)**  **Other signs in ET PKU patients**  - Hyperreflexia: 45% (5/11)  - Clumsiness: 9% (1/11)  **Other signs in LT PKU patients**  **-** Hyperreflexia: 36% (4/11)  - Clumsiness: 36% (4/11)  **-** Hypotonia: 9% (1/11)  **-** Strabismus: 18% (2/11) | **Concurrent blood Phe** in PKU patients without tremor: 632 (221) µmol/L; with tremor: 511 (249) µmol/L  **IDC** in PKU patients without tremor: 550 (411) µmol/L; with tremor: 1174 (715) µmol/L  **IDC** in ET PKU patients without tremor: 386 (118) µmol/L; with tremor: 408 (64) µmol/L  **IDC** in LT PKU patients without tremor: 1404 (305) µmol/L; with tremor: 1653 (441) µmol/L |
| --- | --- | --- | --- | --- |
| Pietz  (1999) | **6 ET AwPKU**  Mean age: 28 yrs  (range: 26-30 yrs) | On diet: 4 patients  Off diet: 2 patients | Tremor in two patients still on diet | Blood Phe in last 3 yrs ranged between 830-1357 µmol/L |
| Pitt  (1971) | **53 UT PKU patients**  Age not specified | All patients were untreated | **Neurological symptoms in 65% (31/48) patients:**  - Epilepsy: 23% (11/48)  - Ataxia: 4% (2/48)  - Tremor: 8% (4/48)  - Upper motor neuron disease and quadriplegia/parapalgia: 25% (12/48) | Mean blood Phe: 1659 (range: 908-2161) µmol/L |
| Pitt  (1991) | **46 UT AwPKU**  Mean age: 40 yrs  (range: 29-72 yrs) | All patients were untreated | Epilepsy: 26% (12/46)  Spasticity: 9% (4/46)  Facial palsy: 2% (1/46)  Deterioration in comparison to Pitt (1971) in three patients (ataxia, tremor, spasticity) | Mean blood Phe: 1180 (range: 784-1950) µmol/L |
| Thompson  (1990) | **4 ET AwPKU**  Age at onset of neurological symptoms: 13-20 yrs  **3 LT AwPKU**  Age at onset of neurological symptoms: 15-25 yrs | Dietary management was withdrawn in mid to late childhood (between 7-18 yrs) in all patients, except one ET | **ET AwPKU**  - Ataxia: 2/4  - Epilepsy: 2/4  - Hyperreflexia: 1/4  - Paraparesis: 1/4  - Quadriparesis: 1/4  **LT AwPKU**  - Epilepsy: 1/3  - Dystonia: 2/3  - Tremor: 1/3  - Sensory changes: 1/3  - Paraparesis: 1/3  - Quadriparesis: 2/3 | - |
| van Vliet  (2019) | **16 LT patients with PKU** | All patients were late treated (but escaped intellectual disability)  Age at diagnosis ranging between 8 yrs and 10 months to 52 yrs | - Visual symptoms: 3/16  - Tremor: 3/16  - Parkinson features: 1/16  - Rigidity: 1/16  - Delayed development: 3/16  - Polyneuropathy: 1/16  - Involuntary contraction in facial muscles: 1/16  - Seizures: 2/16  - Spastic paraplegia: 1/16 | Blood Phe > 1200 µmol/L |
| Villasana  (1989) | **2 AwPKU**  Aged 18 and 28 yrs | One patient initiated diet at infancy and discontinued after age 6 yrs but resumed diet in adulthood  One patient initiated diet at age 3 and discontinued after age 12 | **ET patient**  - Tremor  **LT patient**  - Rigidity  - Seizures  - Spastic dysarthria  - Tremor  - Spasticity  - Brisk tendon reflexes | **Blood Phe ET patient:** 1659 µmol/L  **Blood Phe LT patient**  - Before diet: 1078 µmol/L  - On diet 2 months: 908 µmol/L  - On diet 3 months: 218 µmol/L |
| Waisbren  (2017) | **9 ET AwPKU**  Mean age: 29 (4) yrs | On diet: 7 patients  Relaxed diet: 1 patient  Off diet: 1 patient  Four patients discontinued diet in middle childhood | **No neurological symptoms in 67% (6/9) of patients**  **Other patients:**  - Tremor: 2/9 (both on diet; one patient discontinued diet, one patient did not discontinue diet)  - Brisk reflexes: 1/9 (on diet but discontinued in middle childhood) | Mean blood Phe: 918 (373) µmol/L (range: 277-1512 µmol/L) |
| Çelic  (2018) | **10 LT AwPKU**  Aged between 19-36 yrs | Diagnosis between 3.5 months to  12 yrs | **Patients with epilepsy features were selected**  **Other neurological symptoms:**  - Tremor: 1/8  - Nystagmus: 1/8  - Ataxia: 1/8  - Spastic tetraparesis: 1/8  - Spasticity: 1/8  - Hyperreflexia: 1/8 | - |

Abbreviations: AwPKU: adults with phenylketonuria; DM: diabetes mellitus; ET: early treated; GP: general population; HC: healthy control; LT: late treated; Phe: phenylalanine; MRI: magnetic resonance imaging
