# Supplementary material for: Characterisation and differential diagnosis of neurological complications in adults with phenylketonuria: literature review and expert opinion
Source: J Neurol. 2023 Apr 20;270(8):3675–87. doi: 10.1007/s00415-023-11703-4 (PMC10345006; doi:10.1007/s00415-023-11703-4)
Supplement: Supplementary file 2 — Supplementary file2 (PDF 27 KB) [file 415_2023_11703_MOESM2_ESM.pdf]

Phenylketonuria (PKU)

a treatable cause of neurological symptoms

Background

Complex unexplained neurological and/or psychiatric clinical manifestations in adults can be caused by PKU and other rare inherited metabolic disorders.

PKU is a rare inherited metabolic disorder with neurological manifestations if left untreated.[1] Severe disabilities in patients with PKU can be prevented through the initiation of a low phenylalanine (Phe) diet from diagnosis at newborn screening throughout childhood and adolescence.[2,3] As it was historically assumed that PKU only needed to be treated in childhood, many adults have been lost to follow-up and some have almost 'forgotten' about PKU. However, today it is known that PKU requires lifelong treatment, with patients who have discontinued treatment being at risk for developing various neurological and psychiatric symptoms.

These symptoms are potentially reversible upon resuming or initiating treatment.

Clinical findings

Neurological symptoms can occur immediately or progressively over several months to years upon treatment discontinuation. Importantly, adult patients who have been lost to follow-up may not attribute these symptoms to PKU.

[1] Pietz J Curr Opin Neurol 1998;11(6):679-88
[2] Vockley J, et al. Genet Med 2014;16(2):188-200
[3] van Wegberg AMJ, et al. Orphanet J Rare Dis 2017;12(1):162

Phenylketonuria (PKU)
a treatable cause of neurological symptoms

PKU should be considered in the differential diagnosis for patients presenting with a combination of symptoms from one or more of the following areas

Motor symptoms

- Ataxia
- Tremor
- Hyperreflexia
- Spastic paraparesis
- Stereotypies
- Tics

Cognitive symptoms

- Cognitive decline
- Executive dysfunction

Psychiatric symptoms

- Depression
- Mood swings
- Anxiety disorders
- OCDs

Additional symptoms

- Seizures
- Loss of visual acuity
- Sensory manifestations
- Headache
- Eczema-like skin changes

Differential diagnosis

1. Medical history

- Previous illnesses/findings
- Early childhood development
- Family health history
- Cognitive symptoms
- Psychiatric symptoms
- Questions to the patient†
  - Do you have/have you previously had PKU?
  - Did you follow a specific diet in childhood?
  - Were you allowed to eat protein as a child?
  - When did you start school?
  - What is your highest level of education?

2. Examinations‡

- Blood Phe (dried blood spots or EDTA blood)
- Neurological examination with focus on movement disorders
- Neurocognitive testing (MoCA)
- Psychiatric examination
- MRI
- EEG
- Evoked potentials

† Historically, treatment was discontinued at the age of 12 to 18 years. ‡ Examinations are symptom dependent. EEG: Electroencephalogram; MoCA: Montreal Cognitive Assessment; MRI: Magnetic Resonance Imaging; OCDs: Obsessive Compulsive Disorders
